# Rethinking mental health care provided to migrants and refugees; a randomized controlled trial on the effectiveness of Value Based Counseling, a culturally sensitive, strength-based psychological intervention

**DOI:** 10.1371/journal.pone.0283889

**Published:** 2023-03-31

**Authors:** Tahereh Mina Orang, Inge Missmahl, Maryam Gardisi, Ulrike Kluge

**Affiliations:** 1 International Psychosocial Organisation (IPSO), Berlin, Germany; 2 Klinik für Psychiatrie und Psychotherapie, Charité Universitätsmedizin Berlin, Campus Mitte, Berlin, Germany; 3 Berliner Institut für empirische Integrations- und Migrationsforschung (BIM), Humboldt Universität zu Berlin, Berlin, Germany; Columbia University Mailman School of Public Health, UNITED STATES

## Abstract

**Background:**

Despite traumatic experiences and persistent psychosocial stressors, many refugees and migrants display resilience and strength in the midst and aftermath of hardships. ‘Value Based Counseling’ (VBC), a low-threshold, short-term and culturally sensitive psychological intervention avoids the stigmatization and pathologization of mental health problems, and, in line with latest research calling for a rethink of mental health care for migrants and refugees, focusses on the resilience and resources of clients.

**Method:**

This pragmatic, assessor-blinded randomized controlled trial employed a pre-post control group design to assess the effectiveness of VBC in the development of psychological assets. Refugees and migrants aged 18 or above were randomly assigned to either VBC sessions delivered by counselors matched with their clients according to gender and native language, or to a waiting list.

**Results:**

Per protocol and intention-to-treat (ITT) analyses revealed that compared with participants in the waiting-list group (n = 50), the VBC group (n = 53) experienced a greater improvement in resilience (adjusted difference 11.59, 95% CI 8.35 to 14.84, effect size .49, *p <* .*001*) and perspective taking (adjusted difference 3.98, 95% CI 2.12 to 5.84, effect size .39, *p <* .*001*) after four sessions on average. These positive results remained consistent until a 3-month follow-up assessment within the VBC group.

**Conclusion:**

VBC with a focus on personal resources in the Here and Now, and with a culturally sensitive approach, helps clients exposed to persistent psychosocial stressors to develop strength and to increase agency over their lives.

## Introduction

Considerable mainstream emphasis on pathology-based and trauma-oriented mental health assessments and interventions have paid little attention to potential resources and capabilities that migrants and refugees possess [1–4]. Recent ongoing research has aimed to rethink mental health care of migrants and refugees which has resulted in the employment of non-stigmatizing interventions encouraging clients to reflect on their human values and cultural strengths in the process [2, 5–7]. The present research focusses on the contribution of Value Based Counseling (VBC) to this new direction as a resource-oriented, culturally sensitive intervention. The effectiveness study focusses on improvements in the resilience and perspective taking of migrants and specifically refugees resettled in Germany.

Despite post-migration psychosocial stressors, pre-migration and in-transit trauma and pressure, many migrants and refugees show resilience and strength in the midst and aftermath of hardships, and possess personal, familial, cultural, and community resources. Fast growing research frequently reports high levels of psychological resilience in migrant populations worldwide [8–15]. Personal resilience is defined by a personal sense of confidence, self-efficacy, successful adaptation, positive functioning and the self-belief that one can influence one’s own life and handle or cope with chronic or acute adversities [16–18]. Resilience is increasingly identified as a key potential protective factor against the development of psychological distress and mental health problems such as depression, anxiety and posttraumatic stress disorder in migrant and refugee populations [15, 16, 19] and is significantly associated with positive outcomes such as self-esteem, self-awareness, optimism, self-efficacy, meaning making, and quality of life [5, 6, 9, 20–25]. Previous research shows that resilience-based and resource-oriented interventions improve adaptive behaviors and functionality through an improvement of sense of control, meaning making, sense of purpose, self-awareness, and personal growth [3, 26]. Several different psychological interventions managed to strengthen resilience of migrants and refugees by providing social support, coping skills, and culturally relevant strategies [5, 17, 18, 27].

Perspective taking, an important personal social skill, is particularly relevant to migrants’ experiences of adjustment and integration into host communities, as it is frequently associated with an improvement of social bonds and prosocial behavior [28–32], successful conflict resolution [33, 34], reduction of aggressive behavior [28, 33, 35] and prejudice [36, 37], and results in better mental health [38–40]. Perspective taking is considered to be the cognitive component of empathy, as it enables an individual to “understand others’ inner states and perspectives, and imagine different viewpoints beyond one’s own” [29, 41]. Current evidence shows that interventions can stimulate empathy and perspective taking [28, 31, 32, 35].

The counseling approach used in ‘Value Based Counseling’ (VBC), a low-threshold, short-term and culturally sensitive psychological intervention, goes back to the need to address mental health needs of people living in a context of continuous and ongoing psychosocial stress in Afghanistan in 2005 [42]. When large numbers of refugees began to arrive in Germany in 2015, the approach was used to empower migrants and refugees resettled in Germany, as it corresponds well with the specific needs of mobile, culturally heterogeneous populations like migrants and refugees. Two recent studies investigating the effectiveness of VBC in this context revealed significant symptom reductions with medium to large effect sizes [43, 44].

VBC is based on the core concept of a salutogenic approach, i.e. the sense of coherence (SOC) comprising the constructs of comprehensibility, manageability and meaningfulness [45]. VBC hypothesizes that an enhancement of self-efficacy, meaning making, and awareness of emotions in self and others can activate personal and cultural resources and improve resilience [46]. A full description of the VBC method is published in German [47].

VBC adopts Albert Bandura’s conceptualization of perceived self-efficacy as “beliefs in one’s capabilities to produce given attainments” [48]. To support self-efficacy, VBC limits the exploration of symptoms or the presenting problem to the Here and Now, and focusses on empowering the client by improving their agency. This approach is supported by resilience studies which frequently report that a focus on the Here and Now rather than a deeply-rooted pathology or past traumatic experiences is a source of resilience for migrants and refugees, which helps them to cope with ongoing difficulties [3, 4, 12, 26, 49].

Furthermore, emphasizing the importance of meaning making and self-awareness in the counseling process, VBC encourages clients to connect to their personal and cultural values, strengths and resources through a non-directive but carefully structured conversation with a counselor who shares their language and cultural background. This ideally enables the counselor to recognize relevant triggers, to understand their meaning and to base appropriate action on emotional judgement. A shared deep understanding of the client’s inner situation and living conditions can increase the client’s self-awareness, which, in turn, provides a basis for improving their sense of self-control and self-efficacy [6]. Furthermore, self-awareness is considered to be an important prerequisite for the development of self-knowledge and healthy self-regulation [50], which, in turn, can increase self-compassion, enhance resilience, and facilitate adaptive change [22, 23, 50, 51],

In summary, the growing need for psychological interventions with a resource-oriented, culturally sensitive approach within migrant populations stimulated the present research to examine whether VBC can improve resilience and perspective taking in migrants and refugees resettled in Germany. We also investigated whether improvements within the VBC group lasted for a period of three months.

## Materials and methods

### Trial design

The data presented in this paper is drawn from a RCT study project entitled “Promoting the resilience of refugees and migrants–effectiveness study on the use of native-language counselors” approved by the Ethical Review Board of the Charité (Nr. EA1/034/18). The trial was registered in the German Clinical Trials Register (DRKS00016867). The study project adopted a pre-post design with a waiting list group as a control group and a three-month follow-up to assess the effectiveness of Value Based Counseling (VBC) and its long-term effects on the mental health of migrant and refugee populations in Germany. Participants were randomized with a 1:1 allocation ratio to either a group that received Value-based Counseling after a pre-test (VBC-Group) or a waiting list control group. The present paper presents the data collected on variables of resilience and perspective taking. Two recent papers which focused on further data collected for the study focused on the clinically meaningful reduction of depression and PTSD symptoms, perceived stress, anxiety, and somatic complaints experienced by participants in the VBC group after an average of four counselling sessions [43, 44].

### Setting & local team

International Psychosocial Organization (Ipso gGmbH) conducted the recruitment, counselling interventions and follow-up assessments at an Ipso Care Center located in Berlin. All the counseling sessions, baseline and follow-up diagnostic assessments were conducted by a group of 18 experienced counselors working on a salary basis. They had undergone a one-year full-time training in VBC and received weekly regular technical supervision for their counseling by trained supervisors. Counselors documented every case and every session. They further received a three-day training workshop and regular supervision by an experienced doctoral-level clinical researcher, who was specialized in work with migrants and refugees, to carry out structured interviews based on questionnaires, as not all participants were sufficiently literate to fill in the questionnaires themselves. The tasks of assessing and of counseling a client were assigned to different counselors, based on their availability. Clients and counselors were matched in terms of gender, language and cultural background. Counseling sessions were conducted in Arabic, Dari, Farsi, Kurdish, French, German, Maninka, Pular, or Sousou. S1 File shows a full description of the one-year VCB training course.

### Sampling procedure

Participants had been made aware of the services of the Ipso Care Center through different channels such as advertisements, referrals from health care professionals and social workers, and word of mouth. Those who showed interest in being counseled received verbal and written information on the study and conditions for participation. An information sheet presented details in Farsi, Arabic, French, English, and German as well as contact details of the study coordinator. Participants had at least ten days to read the information sheet, to contact the coordinator for further questions and to decide whether they wanted to participate in the study. If they did not agree, they could proceed with the regular counseling service.

**Fig 1:**
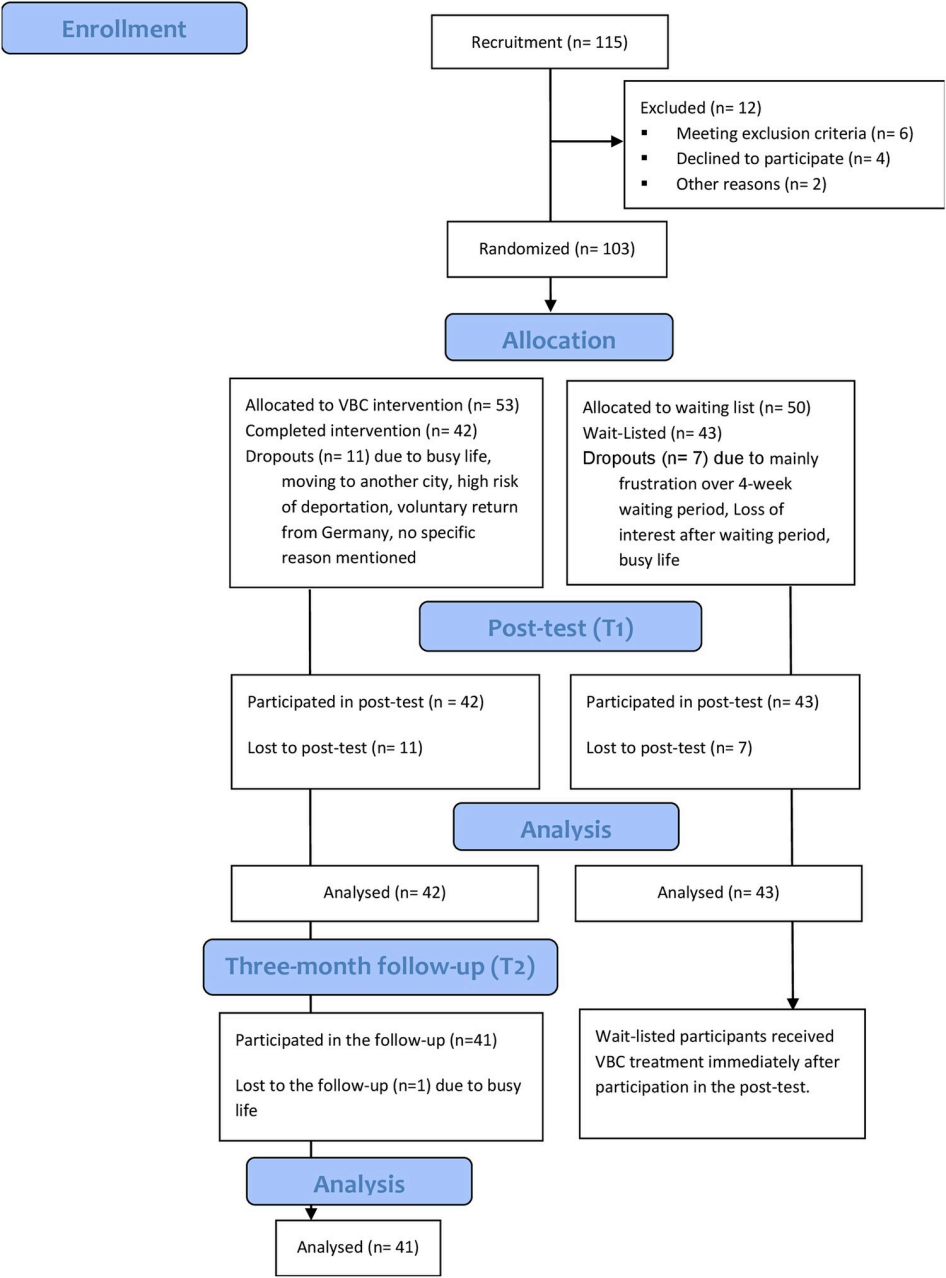
CONSORT flow diagram of the study.

The inclusion criteria were help-seeking migrants, including refugees, and being of age (18 years or above). Exclusion criteria were acute suicidality, acute psychoses, current involvement in psychological treatment, and acute substance dependence. Out of 115 help-seeking people, six met the exclusion criteria following use of appropriate questionnaires (MINI suicidal sub-scale, institutional self-constructed screening sheet) and were referred to appropriate resources, and another six declined to participate in the study for different reasons and made use of the regular counseling service (Fig 1).

After recruitment, participants were randomly allocated to a counseling or a waiting list. Randomization was based upon the sequence of the participants’ arrivals in the center, i.e. the first participant was allocated to the group receiving counseling immediately (VBC group), the second to the waiting list (Waiting-list group) and so forth. The participants were not aware of the allocation procedure handled by the study coordinator. Due to ethical concerns and in line with Ipso policies, the waiting period was limited to four weeks. A few randomized participants in the waiting list group (n = 3) who had not fallen under the exclusion criteria but required urgent counselling were reassigned to the counseling group for ethical reasons. After completion of the consultation in the VBC group, the posttest measurement took place within ten to 14 days. The same set of post-test questionnaires was submitted to the control group after a one-month waiting period (posttest). The participants in the VBC group participated in a further follow-up assessment after three months. Written informed consent was obtained from all participants for inclusion in the study.

### Participants

We recruited 103 participants between March 2018 and May 2019. They were 18 to 62 years old and had been born in the following countries: Iran (n = 40; 38.8%), Afghanistan (n = 22; 21.4%), Syria (n = 22; 21.4%), Guinea (n = 8; 7.8%), Iraq (n = 5; 4.9%), Lebanon (n = 2; 1.9%), Burkina Faso (n = 1; 1%), Egypt (n = 1; 1%), Palestine (n = 1; 1%), and Yemen (n = 1; 1%). Fifty-eight percent of the participants were Muslims, 10% Christians, 1% had other religions (Yazidi), and 31% did not state any religious background. Of the 103 participants, 65% had an asylum-seeking status with a temporary identification document, 24.3% had a temporary residence permit, and few either were EU citizens (2.9%), naturalized Germans (3.9%), or without a legal residence permit (3.9%). Participants had lived in Germany for 45 months on average, ranging from 1 to 480 months. More than half had attended school for approximately 12 years (school diploma; 55.3%), and roughly two thirds did not work at the time of the pretest interview (68.9%). S1 Table presents the sociodemographic profile of the participants across the two study groups.

### Intervention

#### Value Based Counseling (VBC)

VBC is a manualized intervention in two parts. In the first part (Steps 1–3), client and counselor develop a narrative of the client’s biography to develop a mutual understanding of the inner situation of the client in the Here and Now. They seek to understand which psychosocial stressor triggered the vulnerability of the client expressed in a symptom or problematic behavior. In the second part (Steps 4–6), client and counselor focus on the Here and Now of the client with the aim to activate the client’s personal, family and cultural resources and to enable them to define a way forward which allows the client to deal with the psychosocial stressor in a meaningful way, improving their daily functionality with the help of interventions that support the way forward defined by the client. In this context, it is important for the client to improve their self-effectiveness. In the last step, the client’s cognitive and emotional process is reviewed and conceptualized as a resource for personal challenges the client may face in the future. This improves the client’s confidence in their own ability to cope with future challenges and thus improves their resilience further.

*Step 1*: *Understanding the symptom or presenting problem*. Development of a shared understanding of the client’s symptoms or presenting problem in terms of intensity, duration and frequency. Exploration of how the client’s daily functioning is affected by the symptom/problem and by the thoughts and emotions triggered by them. The aim is to understand the meaning of the symptom/problem in the context of the client’s biographic vulnerability and sociocultural context, and not to give a diagnosis.

*Step 2*: *Understanding the psychosocial stressor*. Contextualization and development of a shared understanding of psychosocial stressor; analysis of the human values of the individuals involved in the stressor (involves perspective taking), of the impact of the stressor on the client, and the connection between stressor and symptom.

*Step 3*: *Identification of the dominant feeling tone*. Development of an understanding of the dominant feeling tone of the client which hinders daily functioning most. The connection between the feeling tone and the personal vulnerability of the client is explored. This requires a shared empathetic understanding between counselor and client. Unconscious identification with the dominant feeling tone such as guilt or shame usually can be made conscious, enabling the client to understand why their feeling tone has become dominant. This may also be the moment to include a psychoeducational component in the conversation and to give relevant health information if appropriate (e.g., what constitutes PTSD, etc.).

*Step 4*: *Identification of the main complaint*. Identification of the main complaint, based on the dominant feeling tone that impairs daily functionality the most.

*Step 5*: *Addressing the main complaint*. Identification of a solution to the main complaint. The solution must be personal, manageable and meaningful to the client, and designed to increase their sense of coherence and to improve their resilience.

*Step 6*: *Psychological Interventions*. Development of a personal strategy designed to address the main complaint. The VBC method is integrative in that it employs well-evidenced psychological intervention methods such as reframing or detecting automatic thinking as cognitive behavioral techniques, relaxation exercises, or grounding and distancing techniques for trauma symptoms, or behavioral activation. Psychosocial interventions which support this personal strategy are also applied in this step. This includes the reactivation of personal resources or the development of new ones, with the help of homework designed to strengthen these resources, if appropriate.

*Step 7*: *Summary*, *Consolidation and Outlook*. Reflection on, and anchoring of, the process and the insights gained.

### Measures

The Connor-Davidson Resilience Scale (CD-RISC) was employed to estimate resilience among the study participants. Several evaluation studies on various populations with different cultural and linguistic backgrounds demonstrated excellent psychometric properties for CD-RISC-10 as an efficient measurement of resilience [52–56]. It consists of ten items, rated on a 5-point Likert scale from 0 (*not true at all*) to 4 (*true nearly all of the time*) over the course of the past four weeks, with the higher score reflecting greater resilience, and is available in Farsi, Arabic, English, French, and German. The internal reliability for the scale in the present sample was good (Cronbach’s alpha = 0.84).

Perspective taking was assessed using one subscale of the Interpersonal Reactivity Index (IRI). IRI measures affective components (i.e. empathic concern and personal distress) and cognitive (i.e. fantasy and perspective taking) components of empathy, and demonstrated good validity and reliability across studies [57–59]. The perspective taking subscale composed of seven statements, each scored on a 5-point Likert scale, from 0 (*does not describe me at all*) to 4 (*describes me very well*), and a higher sum score reflects greater perspective-taking capacity. As validated Persian and Arabic versions of the scale were not available, a group of psychologists who are Persian or Arabic native speakers translated it from English using the blind back-translation technique. The Cronbach’s alpha for the perspective taking subscale of the present sample was 0.63. The Cronbach’s alpha reliability test indicated that the two reversed coded items (the items Number 1 and 4) had a negative impact on the internal consistency of the subscale. Deleting Item 4 produced Cronbach’s alpha of 0.67. Removing both reverse coded items 1 (M = 2.14, SD = 1.49) and 4 (M = 1.25, SD = 1.31) produced a Cronbach’s alpha of 0.70. As the study participants may have misunderstood the meaning of these two items, we deleted them from the IRI subscale and conducted the between and within-group statistical analyses with the same result. The mean score of the 7-item IRI subscale was 2.31, with a minimum score of 1.25 and maximum score of 2.89 (range = 1.64).

The reported Cronbach’s Alphas for both questionnaires were independent of the language version of the instruments used (Persian, Arabic, French, German, and English).

Sociodemographic data including age, gender, marital status, nationality, language, religion, residence status in Germany, the length of time spent in Germany, type of accommodation, educational level, job situation, the number of family members in Germany, and the financial situation were collected at the end of the first interview session. The VBC counselors who collected the data in the form of structural interviews had been trained for the purpose in a three-day full-time workshop. For interviews to be blind, different counselors were assigned to the pre-test and the follow-up assessments of individual clients.

### Statistical analysis

In order to achieve a reliable estimation of VBC efficacy, we decided on a sample size of one hundred. To calculate this, we performed a power calculation, following the previous VBC study on women seeking help in Afghanistan due to psychosocial stress, which produced an effect size of three for the efficacy of VBC [42]. Due to the short investigation period and based on our previous work experience with migrant and refugee communities in Germany, we expected a low dropout-rate of 10%. Therefore, assuming an effect size of Cohen’s d = 1–1.5 and a dropout rate of 10% between pre-test and posttest, we allocated 40–50 participants to each study group. We used a website (http://sample-size.net/) to estimate the study sample size.

The study hypotheses were examined through IBM SPSS Statistics version 24 using 2x2 mixed design ANOVA, with score change as a two-level within-subject variable (pre- vs. post-test) and the study group as a two-level between-subject variable (VBC vs. waiting list) for each outcome measure. In this regard, we studied the interaction effect between score change and type of study group. Bonferroni correction was used to adjust for multiple testing. Moreover, to examine extended effects of VBC from pre-test to three-month follow-up, repeated measure ANOVA was employed. Mauchly’s Test of Sphericity was significant for resilience (*X*^2^ (2) = 16.59, *p* < .001), and perspective taking (*X*^2^ (2) = 27.75, *p* < .001). Thus, we reported the Huynh-Feldt correction to meet the assumption.

Independent Samples *t* tests and Chi-square tests (*X*^2^) were performed to identify probable significant differences between groups and subgroups regarding the sociodemographic variables. In cases in which more than 20% of the expected frequencies in large contingency tables were below 5, violating the Chi-square test assumption, we reported the Fisher’s Exact Test result. To do follow-up analyses for statistically significant Chi-square tests in the large contingency tables, we performed Pearson Chi-square post hoc tests using adjusted standardized residuals (S2 File). To avoid committing a Type I Error, we adopted the Gardner pairwise post hoc procedure to identify precise *P* values for each cell [60, 61]. To transform non-normal distributions to normal, we employed a two-step approach of data transformation [62].

To compare effect sizes between the two groups, we calculated the effect size r, using F-ratios and degrees of freedom for each interaction effect (score change from pretest to posttest across the two groups). Concerning the VBC group, we performed Cohen’s d for each pair of pre-test and three-month follow-up assessments. The accepted significance level (α) for two-tailed statistical analyses was *p* < .05, *p* < .01, and *p* < .001.

## Results

Of the 103 participants, 53 formed the VBC group and 50 the waiting list. Randomization did not lead to any significant differences between the two study groups regarding the sociodemographic variables (S1 Table) and the initial outcome measures of perspective taking (*t* (101) = - 0.37, *p* = .70]), and resilience (*t* (101) = 1.03, *p* = .30). The total sample had a mean perspective taking score of 16.23 (SD = 5.43) and a mean resilience score of 19.78 (SD = 8.81). The resilience scores ranged from 1 to 39.

### Primary outcomes

Conducting 2x2 mixed design ANOVA analyses, we found that compared with the waiting-list group, VBC clients experienced significant improvement in resilience (effect size .49) and perspective taking (effect size .39) between the pre-test and post-test. Moreover, the three-month follow-up assessments of counseled participants indicated that these improvements in resilience (Cohen’s d = .69) and perspective taking (Cohen’s d = .57) had lasted for that period. Table 1 shows the results of the outcome measures at baseline and follow-ups across the two groups. The results yielded small to medium effect sizes.

**Table 1:** Means & standard deviations, and between-group & within-group effect sizes of the outcome variables by study groups.

Study Groups	Pre-test M (SD)	Post-test M (SD)	Follow-up M (SD)	Between group effect size Pre-test to post-test	Effect Size Cohen’s d Within VBC group Pre-test to 3-month
**Resilience**					
**VBC (n = 53)**	20.66 (8.26)	28.69 (7.44)	29.24 (6.58)	.49	.69
**WL (n = 50)**	18.86 (9.35)	17.10 (9.10)	___		
**Perspective taking**					
**VBC (n = 53)**	16.03 (5.18)	19.54 (4.06)	19.41 (3.60)	.39	.57
**WL (n = 50)**	16.44 (5.73)	15.56 (5.40)	___		

VBC: Value-Based Counselling; WL: Waiting List

The Bonferroni correction for the interaction effects between the type of group and scores of resilience (Bonferroni adjusted difference 11.59, 95% CI 8.35 to 14.84, *p <* .*001*), and perspective taking (Bonferroni adjusted difference 3.98, 95% CI 2.12 to 5.84, *p <* .*001*) showed significant results at post-test. Table 2 shows the statistical values. Furthermore, we found that improvements remained significant in the period from the pre-test interview to the three-month follow-up assessment in the VBC group.

**Table 2:** ANOVA analyses of main effects and contrasts for outcome measures.

Scale	Source	degrees of freedom (df)	F	*P* value
Resilience				
**Mixed design ANOVA** **	Score change* (main effect)	(1,101)	13.53	*p* < .001
	Score Change x Type of Group	(1, 101)	32.96	*p* < .001
**Repeated measure ANOVA** ***	Score change (main effect)	(1.60, 104)	34.90	*p <* .*001*
	Pretest vs. 3-month follow-up	(1, 52)	47.53	*p <* .*001*
Perspective taking				
**Mixed design ANOVA**	Score change (main effect)	(1, 101)	6.58	.012
	Score Change x Type of Group	(1, 101)	18.33	*p* < .001
**Repeated measure ANOVA**	Score change (main effect)	(1.43, 104)	18.15	*p* < .001
	Pretest vs. 3-month follow-up	(1, 52)	25.14	*p* < .001

* Score change presents changes in sum-scores from pretest to posttest in the total sample group

**Mixed design ANOVA (2x2) uses score change as a two-level within-subject variable (pre- vs. post-test) and the study group as a two-level between-subject variable (VBC vs. waiting list)

***Repeated measure ANOVA only demonstrates outcomes within the VBC group.

### Secondary outcomes

We divided resilience scores into ‘low’ resilience (0–29) and ‘moderate to high’ resilience (30–40) according to the categorizations established by the CD-RISC-10 guideline [63]. S2 Table shows frequencies, percentages, mean and standard deviations of resilience total score for the whole sample and the two resilience subgroups across sociodemographic variables.

Due to the non-normal distributions of the baseline resilience scores defined as ‘low’ and ‘moderate-to-high’ resilience according to the Shapiro-Wilk test, we employed a two-step approach of data transformation in order to meet the normality assumption of the main analyses [62]. However, the independent samples *t* tests for both the age (*t* (101) = 0.03, *p* = .97) and the total months resettled in Germany (*t* (15.91) = -0.82, *p* = .42) did not yield significant differences between the two resilience groups even after the data transformation. We performed several pairs of Chi-square tests (*X*^2^) to identify possible significant differences between the low resilience (n = 86 [83.5%]) and moderate-to-high resilience (n = 17 [16.5%]) groups in relation to sociodemographic variables. However, we did not find any significant differences between the two resilience groups, except for the variables of marital status and nationality. There were significant associations between the type of resilience group and marital status (Pearson Chi-square test (*X*^2^); *X*^2^ (2) = 8.65, *p* = .013), and between the type of resilience group and nationality (Fisher’s Exact Test; 14.53, *p*< .001). Post-Hoc testing of Chi-square test (*X*^2^) performed for marital status did not show significant differences in adjusted residuals within the contingency table at the α level of .0083 (.05/6 = .0083). Contingency tables including frequencies and percentages for each sociodemographic variable across the two resilience subgroups are shown in S2 Table.

Finally, we analyzed whether clients in low and moderate to high resilience categories differ in perspective taking. Independent samples *t* test revealed that clients in the moderate to high resilience group showed greater scores on the perspective taking subscale (M = 20.23, SD = 3.68) in comparison to the clients in the low resilience group (M = 15.44, SD = 5.38) (*t* (101) = -3.50, *p*< .001, 95% confidence interval of the difference -7.50 to -2.07).

## Discussion

In this study, migrants and refugees who immediately received Value Based Counseling significantly improved their resilience and perspective taking in comparison to those on a waiting list. The effectiveness of VBC in the present RCT demonstrated by medium effect sizes indicates that resilience could be strengthened through a resource-oriented, culturally sensitive psychological treatment. Improvements lasted for a period of three months. This promising result supports arguments for the inclusion of VBC in health care systems as a salutogenic approach suitable for migrants and refugees.

Comparing the mean score of resilience in the present sample to the ones in previous migrant and refugee studies, we found that our participants had a lower mean score of resilience at baseline [16, 64]. This might be due to the fact that our sample included help-seeking participants who suffered from significant mental health symptoms and daily functionality impairment. After counseling, their mean resilience score was comparable to mean scores among general populations and community samples in the US and other countries [63]. This promising finding is in line with previous findings on psychological interventions that managed to strengthen the resilience of migrants by providing social support, coping skills, and culturally relevant strategies [5, 17, 18, 27]. Therefore, we can conclude that resilience is not static, but rather a dynamic process that can be stimulated through protective external factors and psychological interventions [65].

Matching the language and cultural background of the client and the counselor in VBC is surmised to be an important factor contributing to improved resilience, as it facilitates a quick route to a shared in-depth understanding of cultural norms and traditions and of personal and collective values that influence a clients’ concept of self, their perception of their social environment, and meaning attributed to inner conflicts. Previous research has shown that sociocultural contexts, religious traditions, and spiritual beliefs which influence meaning-making processes modulate pathways to resilience [66]. In addition to well-investigated findings on the significant effectiveness of conducting mental health treatments in a clients’ native language [67], previous research specifically suggests that culture and context determine the definition of resilience, its indicators, and relevance of resilience strategies [68–70].

Recent trends in mental health care provided to migrants and refugees do not only emphasize the importance of the employment of non-pathologizing and non-stigmatizing but resilience-oriented interventions, but also encourage integration of cultural values, strengths and performances as sources of resilience in the process of recovery after difficult life experiences [2, 5, 6, 26, 71]. In addition, growing research draws our attention to post-migration living conditions and psychosocial stressors in the Here and Now of clients’ lives rather than a deeply-rooted pathology or past traumatic experiences, as the former empower migrants and refugees to cope with ongoing difficulties, which in turn, can enhance their resilience [3, 4, 12, 26, 49]. The VBC approach, originally developed for, and practiced in, mental health support in resource-poor settings, avoids pathologizing psychological symptoms which are an expression of intrapsychic or interpersonal conflicts, traumatic experiences, disruptive social environments, or difficult life transitions such as migration or loss of livelihoods. The counselor instead seeks to understand the meaning of the symptom in the given personal and sociocultural context, based on biographic vulnerabilities of the client and triggered by current psychosocial stressors and unresolved stress. The goal is to empower clients by rediscovering strengths and improving coping skills that allow them to manage daily stressors in a personally meaningful way [46, 47].

Finally, the improvement of perspective taking in the present study has promising implications for the social integration of migrants and refugees in host countries, as research frequently shows positive associations between perspective taking and prosocial behavior [28–32], successful conflict resolution [33, 34], reduction of aggressive behavior [28, 33, 35]. and prejudice [36, 37], and improved mental health [38–40]. VBC counselors support perspective taking capacities in clients when they help them to understand the perspective of other people who are involved in a stressful situation, by understanding their values, their perception of the world based on their values, and their motivations. To be able to empathize with others, clients first need to understand their own inner situation and emotional judgment and to recognize their identification with dominant feeling tones such as fear, anxiety, sadness, shame, or guilt [72]. VBC empowers clients to achieve this through an analysis of associated symptoms, thoughts and feelings and of their impact on the clients’ daily functioning. A detailed description of the VBC method has been published elsewhere [47].

### Limitations

Due to limited resources, the present study could not include a long-term follow-up evaluation and an assessment of some important resilience-related factors such as self-efficacy and self-awareness. Moreover, the uniqueness of our study setting and of the intervention itself, although in line with recent research, made it difficult to compare the VBC method to other mental health treatments. The heterogeneity of our sample due to the inclusion of participants with different cultural backgrounds, languages etc. may affect a generalization of our findings.

### Future studies

Future replication studies on the effectiveness of VBC in regard to the improvement of strengths and positive adaptation in inter- and multicultural settings are desirable. Furthermore, an evaluation of the counseling method in regard to meaning making, self-efficacy and self-awareness, which can lead to an improvement of resilience and empathy, would be valuable.

## Conclusion

Value Based Counseling (VBC) with a focus on personal resources in the Here and Now, and with a culturally sensitive approach, helps clients exposed to persistent psychosocial stressors to develop strength and to improve agency over their lives. The evident efficacy of the VBC model in the present study encourages a salutogenic approach to mental health care, especially for populations exposed to persistent psychosocial stressors in general, and for migrants and refugees in particular.

## Supporting information

S1 Table Baseline sociodemographic characteristics of clients divided by groups (n = 103). (PDF)

S2 Table Baseline sociodemographic characteristics across low and moderate-to-high resilience groups (n = 103). (PDF)

S1 File Value Based Counseling training course. (PDF)

S2 File Pearson Chi-square post hoc test outputs for the large contingency tables. (PDF)
