# Supplementary material for: Rethinking mental health care provided to migrants and refugees; a randomized controlled trial on the effectiveness of Value Based Counseling, a culturally sensitive, strength-based psychological intervention
Source: PLoS One. 2023 Mar 31;18(3):e0283889. doi: 10.1371/journal.pone.0283889 (PMC10065247; doi:10.1371/journal.pone.0283889)
Supplement: S1 Table — (PDF) [file pone.0283889.s001.pdf]

S1 Table.

Baseline sociodemographic characteristics of clients divided by groups (n=103)

Baseline Demographics	VBC group (n=53) n (%)	WL group (n=50) n (%)	*p* Value
**Gender**			
Female	22 (21.4)	18 (17.5)	.56
Male	31 (30.1)	32 (31.1)	
**Marital Status**			
Single	23 (22.3)	24 (23.3)	.72
Married/Partnership	22 (21.4)	21 (20.4)	
Divorced/Widowed	8 (7.8)	5 (4.9)	
**Nationality**			
Afghanistan	10 (9.7)	12 (11.7)	
Iran	23 (22.3)	17 (16.5)	.83
Syria	11 (10.7)	11 (10.7)	
Other Arab countries	4 (3.9)	6 (5.8)	
African countries	5 (4.9)	4 (3.9)	
**Religion**			
Muslim	29 (28.7)	31 (30.7)	.44
Christiane	7 (6.9)	3 (3)	
No religion	16 (15.8)	15 (14.9)	
**Residence Status**			
Asylum seeker	37 (35.9)	30 (29.1)	.57
Acquiesce	11 (10.7)	14 (13.6)	
Other	5 (4.9)	6 (5.8)	
**Accommodation**			
Refugee dormitory	27 (26.2)	21 (20.4)	.63
Private apartments	18 (17.5)	21 (20.4)	
Others	8 (7.8)	8 (7.8)	
**Education**			
Elementary to middle school	20 (19.4)	14 (13.6)	.31
Diploma	29 (28.2)	28 (27.2)	
No education	4 (3.9)	8 (7.8)	
**Professional Qualification**			
University degree	21 (20.6)	16 (15.7)	.25
Vocational training	7 (6.9)	13 (12.7)	
Other	11 (10.8)	6 (5.9)	
None	13 (12.7)	15 (14.7)	
**Work**			
No	37 (35.9)	34 (33)	.84
Yes	16 (15.5)	16 (15.5)	
**Income**			
Less than 500 €	29 (29.6)	28 (28.6)	.95
500-1000 €	12 (12.2)	10 (10.2)	
1000-1500 €	10 (10.2)	9 (9.2)	
**Family Members in Germany**			
None	20 (19.4)	20 (19.4)	.84
One or two	10 (9.7)	11 (10.7)	
Three or more	23 (22.3)	19 (18.4)	
**Age:** M (SD)	30.42 (7.79)	30.75 (8.43)	.80
**Time spent in Germany:** M (SD)	33.44 (16.90)	35.20 (23.81)	.66

VBC: Value-Based Counseling; WL: Waiting List
