# Supplementary material for: Rethinking mental health care provided to migrants and refugees; a randomized controlled trial on the effectiveness of Value Based Counseling, a culturally sensitive, strength-based psychological intervention
Source: PLoS One. 2023 Mar 31;18(3):e0283889. doi: 10.1371/journal.pone.0283889 (PMC10065247; doi:10.1371/journal.pone.0283889)
Supplement: S2 Table — (PDF) [file pone.0283889.s002.pdf]

S2 Table.

Baseline sociodemographic characteristics across low and moderate-to-high resilience groups (n=103)

Baseline Demographics<br>n (%)	Whole Sample<br>(n= 103)	Low resilience<br>(n=86)	Moderate to high resilience (n=17)	*P value*
Gender				
**Female**	40 (38.8)	33 (38.4)	7 (41.2)	.82
**Male**	63 (61.2)	53 (61.6)	10 (58.8)	
Marital Status				
**Single**	47 (45.6)	44 (51.2)	3 (17.6)	
**Married/Partnership**	43 (41.7)	34 (39.5)	9 (52.9)	.01*
**Divorced/Widowed**	13 (12.6)	8 (9.3)	5 (29.4)	
Nationality				
**Afghanistan**	22 (21.4)	18 (20.9)	4 (23.5)	
**Iran**	40 (38.8)	35 (40.7)	5 (29.4)	.00**
**Arab countries (e.g. Syria)[1]**	32 (31.1)	30 (34.9)	2 (11.8)	
**African countries[2]**	9 (8.7)	3 (3.5)	6 (35.3)	
Religion				
**Muslim**	60 (59.4)	46 (54.8)	14 (82.4)	
**Christiane**	10 (9.9)	9 (10.7)	1 (5.9)	.10
**No religion**	31 (30.7)	29 (34.5)	2 (11.8)	
Residence Status				
**Asylum seeker/refugee**	67 (65)	54 (62.8)	13 (76.5)	
**Acquiesce**	25 (24.3)	24 (27.9)	1 (5.9)	.08
**Other[3]**	11 (10.7)	8 (9.3)	3 (17.6)	
Accommodation				
**Refugee dormitory**	48 (46.6)	37 (43)	11 (64.7)	
**Private apartments**	39 (37.9)	34 (39.5)	5 (29.4)	.21
**Others (e.g. student dormitory)**	16 (15.5)	15 (17.4)	1 (5.9)	
Education				
**Elementary to middle school**	34 (33)	26 (30.2)	8 (47.1)	
**Diploma**	57 (55.3)	50 (58.1)	7 (41.2)	.37
**No education**	12 (11.7)	10 (11.6)	2 (11.8)	
Professional Qualification				

**University degree**	37 (35.9)	31 (36.5)	6 (35.3)	
**Other (e.g. vocational training)**	37 (35.9)	31 (36.5)	6 (35.3)	.98
**None**	28 (27.2)	23 (27.1)	5 (29.4)	
Work				
**No**	71 (68.9)	59 (68.6)	12 (70.6)	.87
**Yes**	32 (31.1)	27 (31.4)	5 (29.4)	
Income				
**Less than 500 €**	57 (56.4)	49 (58.3)	8 (47.1)	.39
**More than 500 €**	44 (43.6)	35 (41.7)	9 (52.9)	
Family Members in Germany				
**None**	40 (38.8)	31 (36)	9 (52.9)	.41
**One or two**	21 (20.4)	18 (20.9)	3 (17.6)	
**Three or more**	42 (40.8)	37 (43)	5 (29.4)	
Age: M (SD)	30.76 (8.63)	30·77 (8.36)	30·70 (10.15)	.97
Range	18-62			
Time spent in Germany: M (SD)	45.17 (64.08)	41.23 (47.60)	66.12 (118.85)	.42
Range (months)	1-480			

**. Significant difference at the 0.05 level.*

***. Significant difference at the 0.01 level.*

[1.] *Arab countries include participants mostly from Syria (n= 22), and then Iraq (n= 5), Lebanon (n= 2), Egypt (n= 1), Palestine (n= 1), and Yemen (n= 1).*

[2.] *African countries include participants from Guinea (n= 8), and Burkina Faso (n= 1).*

[3.] This category includes naturalized Germans (n= 4), EU citizens (n= 3), and participants without a residence permit (n= 4).
