# Supplementary material for: Rethinking mental health care provided to migrants and refugees; a randomized controlled trial on the effectiveness of Value Based Counseling, a culturally sensitive, strength-based psychological intervention
Source: PLoS One. 2023 Mar 31;18(3):e0283889. doi: 10.1371/journal.pone.0283889 (PMC10065247; doi:10.1371/journal.pone.0283889)
Supplement: S1 File — (PDF) [file pone.0283889.s003.pdf]

S1 File. Value Based Counseling training course.

Ipso's psychological counselors have received a one-year full-time training in VBC which in Germany is certified by DEKRA and therefore eligible for financial support by German job centers as a type of further education, and in Afghanistan is certified by the Ministry of Public Health. Counselors ideally have a professional background in health care or social sciences obtained in their birth country or elsewhere, but in some cases practical experience in social work may suffice. They can speak with clients in their mother tongue such as Dari/Farsi or Arabic, and the counselors have a migrant background that allows them to relate to the life experiences of clients. Peer counseling however is not a guarantee for success but contains the risk of counselor and client jointly descending into despair if the counselor is not emotionally stable enough to cope with repeated exposure to familiar woes of clients such as traumatic experiences. Emotional stability therefore is an important criterion for the selection of trainees. Supervision on a regular basis needs to ensure that this stability does not erode over time.

The training is divided into two phases, the first consisting of 60 days of full-time intensive training. This part is focused on the transfer of knowledge and skills combined with increased self-awareness enabling trainees to internalize a counseling approach based on empathetic understanding and best practices. Morning sessions cover psychological, psychopathological and socio-cultural issues and intervention techniques. In the afternoons, participants form smaller groups and are encouraged to relate the contents of the morning sessions to their own lives, raising their self-awareness. This unique element of the training enables participants to develop a non-judgmental attitude towards others based on empathy. A written exam at the end of the three-month period tests knowledge, and an oral exam is focused on attitudes and practical skills.

Trainees who pass these exams move on to nine months of full-time on-the-job training during which they are closely supervised and receive 46 days of follow-up trainings. These trainings deepen knowledge and improve skills obtained in the first three months, teach case documentation and management, provide opportunities to deal with personal challenges and teach self-care. Supervision includes face-to-face or online sessions on a weekly basis and monitor progress, identify challenges and provide support to the trainees. Final exams consist of a written and an oral exam as well as the documentation of a case discussed with a colloquium.

Qualified counselors are supervised on a weekly basis, both in groups and individually because supervision is essential for the quality assurance of Value-Based Counseling. The sessions provide counselors with the chance to discuss cases they are concerned about and ensure that counselors keep up a sufficient level of self-care in a work environment that confronts them with harrowing experiences of clients on a regular basis.
