# Supplementary material for: Rethinking mental health care provided to migrants and refugees; a randomized controlled trial on the effectiveness of Value Based Counseling, a culturally sensitive, strength-based psychological intervention
Source: PLoS One. 2023 Mar 31;18(3):e0283889. doi: 10.1371/journal.pone.0283889 (PMC10065247; doi:10.1371/journal.pone.0283889)
Supplement: S2 File — (PDF) [file pone.0283889.s004.pdf]

S2 File

Pearson Chi-square post hoc test outputs for the large contingency tables

**2 x 3 contingency table of marital status and two subcategories of resilience**

### Case Processing Summary

| | Cases | | | | | |
|---|---|---|---|---|---|---|
| | Valid | | Missing | | Total | |
| | N | Percent | N | Percent | N | Percent |
| Recoding marital status into 3 categories * ResiliececCATEGORY | 103 | 100.0% | 0 | 0.0% | 103 | 100.0% |

### Recoding marital status into 3 categories * ResilieceCATEGORY Crosstabulation

| | | | ResilieceCATEGORY | | |
|---|---|---|---|---|---|
| | | | Low resilience | Moderate to high resilience | Total |
| Recoding marital status into 3 categories | single | Count | 44 | 3 | 47 |
| | | Expected Count | 39.2 | 7.8 | 47.0 |
| | | % within ResilieceCATEGORY | 51.2% | 17.6% | 45.6% |
| | | Standardized Residual | .76 | -1.71 | |
| | married-in a relationship | Count | 34 | 9 | 43 |
| | | Expected Count | 35.9 | 7.1 | 43.0 |
| | | % within ResilieceCATEGORY | 39.5% | 52.9% | 41.7% |
| | | Standardized Residual | -.32 | .71 | |
| | divorced-widowed | Count | 8 | 5 | 13 |
| | | Expected Count | 10.9 | 2.1 | 13.0 |
| | | % within ResilieceCATEGORY | 9.3% | 29.4% | 12.6% |
| | | Standardized Residual | -.87 | 1.95 | |
| Total | | Count | 86 | 17 | 103 |
| | | Expected Count | 86.0 | 17.0 | 103.0 |
| | | % within ResilieceCATEGORY | 100.0% | 100.0% | 100.0% |

### Chi-Square Tests

| | Value | df | Asymptotic Significance (2-sided) |
|---|---|---|---|
| Pearson Chi-Square | 8.653[a] | 2 | .013 |
| Likelihood Ratio | 8.520 | 2 | .014 |
| Linear-by-Linear Association | 8.536 | 1 | .003 |
| N of Valid Cases | 103 | | |

a. 1 cells (16.7%) have expected count less than 5. The minimum expected count is 2.15.

| adjusted standardized residuals for marital status | adjusted p value |
|---|---|
| 2.54 | .011241 |
| -2.54 | .011241 |
| -1.02 | .305734 |
| 1.02 | .305734 |
| -2.28 | .022525 |
| 2.28 | .022525 |

## 2 x 4 contingency table of nationality and two subcategories of resilience

### Case Processing Summary

| | Cases | | | | | |
|---|---|---|---|---|---|---|
| | Valid | | Missing | | Total | |
| | N | Percent | N | Percent | N | Percent |
| Recodeorigine * ResilieceCATEGORY | 103 | 100.0% | 0 | 0.0% | 103 | 100.0% |

### Re_recodeorigine * ResiliececCATEGORY Crosstabulation

| | | | ResiliececCATEGORY | | |
|---|---|---|---|---|---|
| | | | 1.00 | 2.00 | Total |
| Re_recodeorigine | **Afghanistan** | Count | 18 | 4 | 22 |
| | | Expected Count | 18.4 | 3.6 | 22.0 |
| | | % within ResiliececCATEGORY | 20.9% | 23.5% | 21.4% |
| | | Adjusted Residual | -.24 | .24 | |
| | Iran | Count | 35 | 5 | 40 |

| | | | | | |
|---|---|---|---|---|---|
| | | Expected Count | 33.4 | 6.6 | 40.0 |
| | | % within ResilieceCATEGORY | 40.7% | 29.4% | 38.8% |
| | | Adjusted Residual | .87 | -.87 | |
| | Arab countries | Count | 30 | 2 | 32 |
| | | Expected Count | 26.7 | 5.3 | 32.0 |
| | | % within ResilieceCATEGORY | 34.9% | 11.8% | 31.1% |
| | | Adjusted Residual | 1.88 | -1.88 | |
| | African countries | Count | 3 | 6 | 9 |
| | | Expected Count | 7.5 | 1.5 | 9.0 |
| | | % within ResilieceCATEGORY | 3.5% | 35.3% | 8.7% |
| | | Adjusted Residual | -4.24 | 4.24 | |
| Total | | Count | 86 | 17 | 103 |
| | | Expected Count | 86.0 | 17.0 | 103.0 |
| | | % within ResilieceCATEGORY | 100.0% | 100.0% | 100.0% |

**Chi-Square Tests**

| | Value | df | Asymptotic Significance (2-sided) | Exact Sig. (2-sided) | Exact Sig. (1-sided) | Point Probability |
|---|---|---|---|---|---|---|
| Pearson Chi-Square | 19.385[a] | 3 | .000 | .000 | | |
| Likelihood Ratio | 14.854 | 3 | .002 | .003 | | |
| Fisher's Exact Test | 14.536 | | | .001 | | |
| Linear-by-Linear Association | 2.523[b] | 1 | .112 | .139 | .075 | .034 |
| N of Valid Cases | 103 | | | | | |

a. 2 cells (25.0%) have expected count less than 5. The minimum expected count is 1.49.

b. The standardized statistic is 1.588.

| adjusted standardized residuals for nationality | adjusted p value |
|---|---|
| -.24 | .811158 |
| .24 | .811158 |
| .87 | .382976 |
| -.87 | .382976 |
| 1.88 | .059814 |
| -1.88 | .059814 |
| -4.24 | .000022 |
| 4.24 | .000022 |
